# Supplementary material for: On the driver of blood circulation beyond the heart
Source: PLoS One. 2023 Oct 19;18(10):e0289652. doi: 10.1371/journal.pone.0289652 (PMC10586597; doi:10.1371/journal.pone.0289652)
Supplement: S1 File — (DOCX) [file pone.0289652.s001.docx]

# Supporting Information

## S1 Appendix. IR energy and temperature measurement

### IR energy measurement

To measure the IR energy imparted to the preparation, we used an optical power meter (Newport, 1835-C) coupled with a power detector (Newport, 818T-10). We placed the detector sensor 10 cm from the center surface of the IR bulb. The reading was ~340 mw. The surface area of the sensor is 2.84 cm^2^; thus, the IR energy density was measured as ~120 mW/cm^2^. For comparison, the average annual solar irradiance at the top of the Earth's atmosphere is roughly 139 mW/cm^2^­ [1].

### Temperature measurement

The temperature increase arising from IR treatment was measured with a thermometer (Omega, HH306A) outfitted with a type-K thermocouple. The thermocouple was inserted immediately underneath the surface of the egg yolk. Due to the principle of humane use of animals, the temperature measurements were performed on non-experimental samples, e.g., on abnormally developed or non-developed eggs. The result is in S1 Fig.


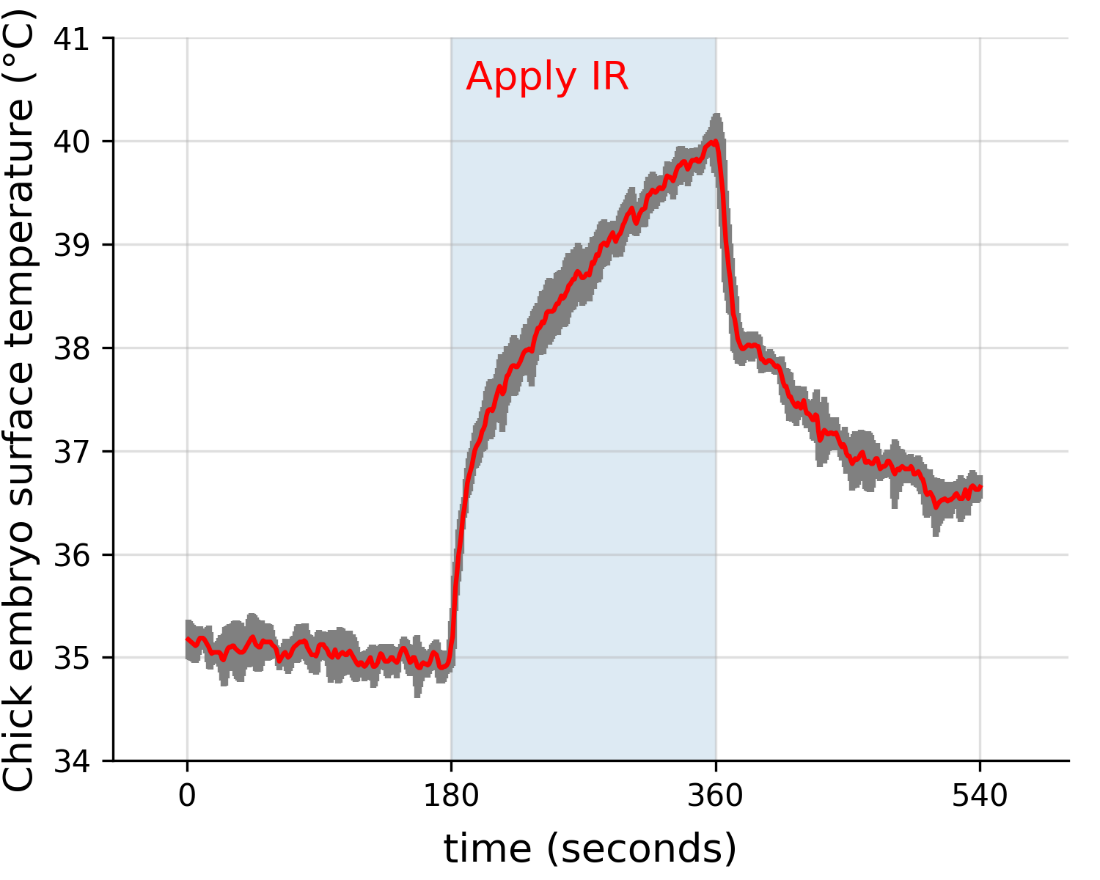


**S1 Fig. Chick embryo surface temperature change resulting from IR radiation.** *n* = 5. Error bars (grey) show standard deviations.

## S2 Appendix. A calculation of surface-area-to-volume ratio of a single blood vessel and a vascular network

Consider a section of a cylindrical blood vessel. The diameter is $D$; the length is $L$; the surface area is $S$; and the volume is $V$.

The surface-area-to-volume ratio of this piece of blood vessel is:

$$\begin{aligned} \frac{S}{V}=\frac{\pi D\cdot L}{\frac{1}{4}\pi D^{2}\cdot L}= \frac{4}{D}\#\left( 1 \right) \end{aligned}$$

Thus, for a single section of blood vessel, the surface-to-volume ratio is inversely proportional to the diameter of the vessel.

Next, consider a vascular network. Regardless of geometric complexity, a vascular network can be considered as the sum of $N$ segments of non-branching blood vessels. Assume the blood vessels are cylindrical, the length of the $i$th segment is $L_{i}$; the diameter of the $i$th segment is $D_{i}$; the surface-area of the $i$th segment is $S_{i}$; the volume of the $i$th segment is $V_{i}$. The surface-area of the vascular bed is $S$, and the volume of the vascular bed is $V$. The surface-area-to-volume ratio of this vascular bed is:

$$\begin{aligned} \frac{S}{V}=\frac{\sum_{i=1}^{N} S_{i}}{\sum_{i=1}^{N} V_{i}}=\frac{\sum_{i=1}^{N} \pi D_{i}\cdot L_{i}}{\sum_{i=1}^{N} \frac{1}{4}\pi D_{i}^{2}\cdot L_{i}}= 4\frac{\sum_{i=1}^{N} {D_{i}L}_{i}}{\sum_{i=1}^{N} D_{i}^{2}L_{i}}\#\left( 2 \right) \end{aligned}$$

Blood vascular network has a hierarchical structure. Within the same hierarchical level, the blood vessels have similar diameters. Assume the average diameter of blood vessels is $D$. From equation (2), we can get the surface-area-to-volume ratio of the blood vessels in a hierarchical level:

$$\begin{aligned} \frac{S}{V}=4\frac{\sum_{i=1}^{N} {DL}_{i}}{\sum_{i=1}^{N} D^{2}L_{i}}=\frac{4}{D}\cdot\frac{\sum_{i=1}^{N} L_{i}}{\sum_{i=1}^{N} L_{i}}=\frac{4}{D}\#\left( 3 \right) \end{aligned}$$

Thus, like a single section of blood vessel, the surface-to-volume ratio at each hierarchical level is inversely proportional to the average diameter of the vessels.

## S3 Appendix. Existence of proton concentration gradients in biological systems

Ideally, it would be best to demonstrate that blood vessels themselves can generate proton gradients. However, due to technical challenges, we could not perform such experiments. Even if we could demonstrate the existence of proton gradients *in vivo*, a problem still exists: the ions/gases dissolved in the blood/surrounding tissues could potentially affect the pH in the region adjacent to vascular wall, making it difficult to demonstrate that a proton gradient can definitively originate from surface-water interaction. Thus, we chose to perform *in vitro* experiments to demonstrate that materials similar to the major component of the vascular wall can generate a proton gradient via surface-water interaction.

Blood vessels are lined with a glycocalyx, a gel-like mesh that has a polysaccharide structure [2]. To mimic the function of the glycocalyx, we used agarose, a polysaccharide material that was previously used as a model to simulate the glycocalyx [3]. Agarose can generate an EZ [4]. Hence, in theory, agarose should be able to generate a proton gradient.

To visualize the expected gradient, we adopted the following procedure. Agarose (Sigma-Aldrich, A9045) comes in powder form. First, we repeatedly dialyzed agarose powder against DI water (obtained from a deionized water system, Barnstead, Nanopure analytical system, D11901), until the conductance of the dialyzing water did not change any more. The purpose of dialysis was to remove any soluble ions, including the ones that might be acidic. We then prepared the agarose hydrogel (1.5% w/v) from the dialyzed agarose powder [4]. After the gel was prepared, we added pH indicator solution (ScienceLab, SLU1951, mixed with DI water at a ratio of 1:10) on the top of the gel. The pH indicator allowed visualization of the proton-rich (acidic) region by its red color. The result is shown below (S2 Fig): the pH indicator showed a proton-rich (acidic) region at the gel/water interface. This proton-rich region showed up almost immediately when the pH indicator solution was injected on top of the agarose gel, and gradually increased in size. Hence, protons, caused by a surface-water (in this case, polysaccharide-water) interaction, are indeed present.


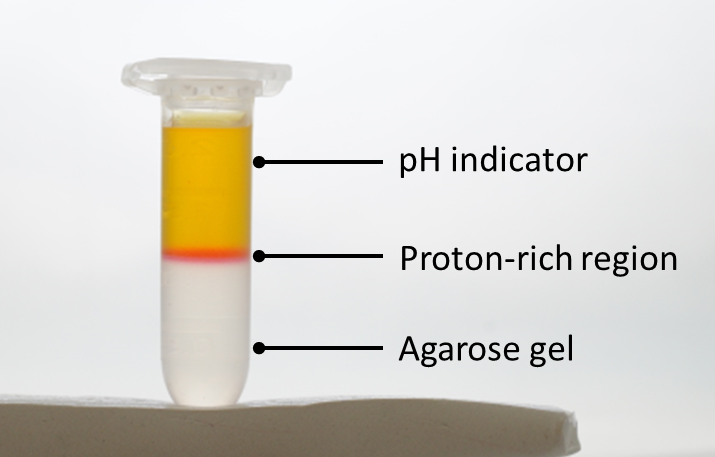


**S2 Fig. Visualization of protons generated from surface-water interaction.** The agarose hydrogel (1.5% w/v) was made from agarose powder that was dialyzed against DI water. A pH indicator solution was poured on top of the agarose gel. At the gel/indicator interface, a region with red color emerges, indicating the presence of protons. This picture was taken five minutes after the pH indicator contacted the surface of the agarose gel.

## S4 Appendix. Quantitative estimation on the flow-driving mechanism

We have been pondering the question of how to describe the flow-driving mechanism quantitatively. In so doing, we have also considered some thermo-driven nanofluid models [5-12]. When it comes to the circulatory system, however, the calculation involves multiple assumptions; hence we are hesitant to draw any firm quantitative conclusions.

That being said, we present preliminary thoughts on the lowest and highest estimates of the energy provided by this flow-driving mechanism: how much does the flow presented in this work contribute to the total energy budget of the blood circulation.

**Lowest estimate**: While the heart was still beating, the blood-flow velocity in anterior/posterior vitelline vein was ~1500 μm/s – the physiological value. After the heart stopped beating, it dropped to ~25 μm/s. Thus, blood vessels are capable of driving at least 1.7% of the physiological blood flow.

After the heart stopped beating, however, the non-pumping heart posed a flow obstacle. It restricts flow. Thus, the aforementioned 1.7% is a conservative estimate. It could be higher. It is worth noting that the material-exchange-driven flow mechanism needs pressure to operate. When the heart stops beating and there is no longer any ventricular pressure, this mechanism can no longer operate. While we could not quantify the magnitude of this flow component, we are confident to say that the sum of the material-exchange-driven flow and the surface-water-interaction-driven flow amounts to more than 1.7% of the total flow.

**Highest estimate**: Blood cells must be driven through capillaries that are sometimes narrower than the RBCs that need to pass through. Therefore, those cells must get squeezed during their passage. Videos confirm erythrocyte bending. Considering the energy required to bend each blood cell, the overall energy requirement could well exceed the capability of the heart (power = ~1.5 W). Hence, the needed energy must come from elsewhere.

Calculation of that energy requires multiple assumptions, which means that definitive conclusions on the relative contribution of the vascular mechanism may be difficult to draw. Nevertheless, the possibility exists that the vessels may contribute a very substantial fraction of the total driving energy. Additional experiments of different nature will be required to estimate just how much.

In sum, we are eager to determine just how much the vessel-driving system may contribute, but at present, too many unknowns exist to allow us to draw meaningful conclusions. It could vary between 1.7% of total and a very major percentage. Any attempt to estimate the amount without additional rigorous experimental study would be highly speculative, and potentially misleading.

## S5 Appendix. Additional analysis on the direction of the surface-induced flow

To reiterate, the driving force behind surface-induced flow should be the substance-concentration gradient, rather than the absolute quantity of the substance.

Consider a cylindrical tube with radius $R$ and length $L$, where substance $X$ can enter or exit the tube through its surface at a rate of amount $A$ per unit surface area per unit time. In this piece of tube, we define the concentration of substance $X$, denoted as $c_{X}$, as the quantity of the substance, $n$*,* divided by the volume of the tube, $V$. Assuming the initial substance concentration is $c_{X(t=0)}$, then at a given time interval $\Delta t$, the concentration of substance $c_{X}$ can be expressed as the sum of the initial concentration and the change of concentration:

$$c_{X}{=c_{X(t=0)}+\Delta c}_{X}=c_{X(t=0)}+\frac{\Delta n}{V}=c_{X(t=0)}+\frac{A\cdot2\pi R\cdot L}{\pi R^{2}\cdot L}\Delta t=c_{X(t=0)}+2A\Delta t\frac{1}{R}$$

Considering the model depicted in Fig 8, where a narrow tube is connected to a wide tube, let us assume that substance $X$ enters the tube (e.g., *A* is positive) and the initial substance concentration is the same in both tubes. Based on the equation above, at a given time interval $\Delta t$, the concentration difference between the narrow section and the wide section is given by:

$${c_{X}}_{narrow}-{c_{X}}_{wide}=2A\Delta t\left( {\frac{1}{R}}_{narrow}-{\frac{1}{R}}_{wide} \right)<0$$

Thus, the substance concentration in the narrower section will be greater than that in the wider section.

Assuming the flow of the substance in the longitudinal direction is caused by diffusion, the direction of the flow should follow the direction of the concentration gradient - from high concentration to low concentration. Since the substance concentration is higher in the narrow tube, the direction of the longitudinal flow should be from narrow to wide.


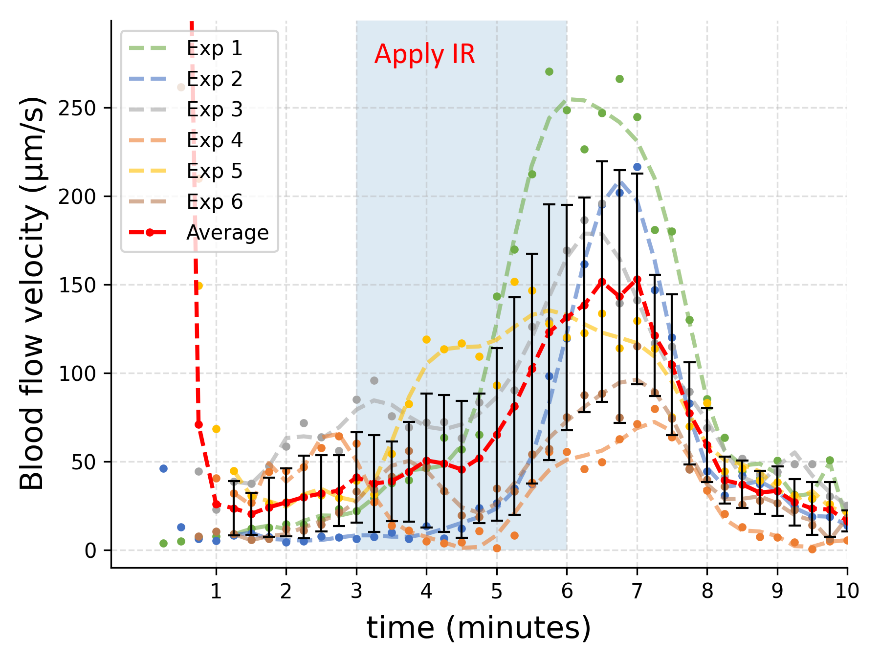


**S3 Fig. Same as Fig 5, with the data for each experiment plotted.**


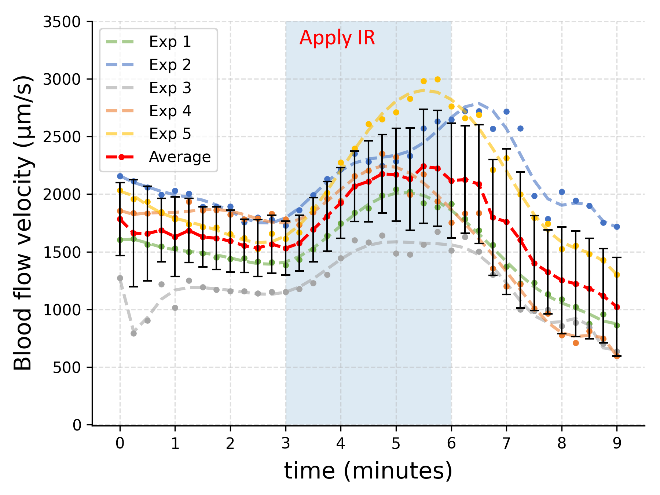


**S4 Fig. Same as Fig 6, with the data for each experiment plotted.**

## References

1. Coddington O, Lean JL, Pilewskie P, Snow M, Lindholm D. A Solar Irradiance Climate Data Record. Bulletin of the American Meteorological Society. 2016;97(7):1265-82.

2. Van Teeffelen JW, Brands J, Stroes ES, Vink H. Endothelial glycocalyx: sweet shield of blood vessels. Trends Cardiovasc Med. 2007;17(3):101-5.

3. Mattern KJ, Nakornchai C, Deen WM. Darcy permeability of agarose-glycosaminoglycan gels analyzed using fiber-mixture and donnan models. Biophys J. 2008;95(2):648-56.

4. Li Z, Pollack GH. Surface-induced flow: A natural microscopic engine using infrared energy as fuel. Science Advances. 2020;6(19):eaba0941.

5. Rasool G, Wakif A. Numerical spectral examination of EMHD mixed convective flow of second-grade nanofluid towards a vertical Riga plate using an advanced version of the revised Buongiorno’s nanofluid model. Journal of Thermal Analysis and Calorimetry. 2021;143(3):2379-93.

6. Wakif A, Abderrahmane A, Guedri K, Bouallegue B, Kaewthongrach R, Kaewmesri P, et al. Importance of exponentially falling variability in heat generation on chemically reactive von kármán nanofluid flows subjected to a radial magnetic field and controlled locally by zero mass flux and convective heating conditions: A differential quadrature analysis. Frontiers in Physics. 2022;10.

7. Wakif A, Animasaun IL, Khan U, Shah NA, Thumma T. Dynamics of radiative-reactive Walters-b fluid due to mixed convection conveying gyrotactic microorganisms, tiny particles experience haphazard motion, thermo-migration, and Lorentz force. Physica Scripta. 2021;96(12):125239.

8. Wakif A, Animasaun IL, Satya Narayana PV, Sarojamma G. Meta-analysis on thermo-migration of tiny/nano-sized particles in the motion of various fluids. Chinese Journal of Physics. 2020;68:293-307.

9. Wakif A, Animasaun IL, Sehaqui R. A Brief Technical Note on the Onset of Convection in a Horizontal Nanofluid Layer of Finite Depth via Wakif-Galerkin Weighted Residuals Technique (WGWRT). Defect and Diffusion Forum. 2021;409:90-4.

10. Wakif A, Chamkha A, Thumma T, Animasaun IL, Sehaqui R. Thermal radiation and surface roughness effects on the thermo-magneto-hydrodynamic stability of alumina–copper oxide hybrid nanofluids utilizing the generalized Buongiorno’s nanofluid model. Journal of Thermal Analysis and Calorimetry. 2021;143(2):1201-20.

11. Wakif A, Shah NA. Hydrothermal and mass impacts of azimuthal and transverse components of Lorentz forces on reacting Von Kármán nanofluid flows considering zero mass flux and convective heating conditions. Waves in Random and Complex Media. 2022:1-22.

12. Wakif A, Zaydan M, Alshomrani AS, Muhammad T, Sehaqui R. New insights into the dynamics of alumina-(60% ethylene glycol + 40% water) over an isothermal stretching sheet using a renovated Buongiorno's approach: A numerical GDQLLM analysis. International Communications in Heat and Mass Transfer. 2022;133:105937.
