# Supplementary material for: On the driver of blood circulation beyond the heart
Source: PLoS One. 2023 Oct 19;18(10):e0289652. doi: 10.1371/journal.pone.0289652 (PMC10586597; doi:10.1371/journal.pone.0289652)
Supplement: Attachment — Submitted filename: Referee_Report.pdf [file pone.0289652.s007.pdf]

Report on manuscript # PONE-D-22-35747 "On the Driver of Blood Circulation Beyond the Heart" by Zheng Li and Gerald H. Pollack.

The paper reports evidences of the existence of a flow-driven mechanism using infrared energy. Experiments are performed using a three-day-old chick-embryo model. The authors demonstrate that when infrared is applied the blood flow increases and when infrared energy is decreases the flow also decrease. Furthermore, when the heart of the chicken embryo is stopped the blood keeps flowing when infrared energy is applied.The experiments are nicely performed and the results reported are relevant. However the models proposed are mostly speculative and do not contribute to revel the mechanism leading to the blood flow when infrared energy is applied. In my opinion, a more elaborate mechanism should be proposed to be able to publish the manuscript in PLOS.

Some comments are given below,

1. When the embryo heart was stopped, IR energy was able to fuel the blood flow. In the experiments reported, IR was applied for a few minutes. I wonder how long the IR energy is able to generate a blood flow after the heart stops.

2. The authors claim that surface-induced flow is the mechanism, triggered by IR energy, that explain the postmortem blood flow. Two kinds of surface-induced flows are proposed: i) material exchange across the vascular walls and ii) water-surface interaction. The explanations of both mechanisms are very speculative and more precise and quantitative description should be provided. To say that "Chemical potential energy is converted in kinetic energy" is ambiguous, can the chemical potential energy be estimated? how is the energy converted in kinetic energy?

3. I am not sure that the mechanism described in figure 4A, in a tube with uniform physico-chemical surface activity, is correct. If we applied the mass conservation equation to the narrow and the wide section we obtain that the concentration is the same in both sections, since the flowrate is the same. In fact, applying mass conservation to the narrow section,

$$m_{in} + m_s = m_{on}$$

where m_{in} is the mass flowrate at the input, m_s the mass flowrate of the substance entering the control volume and m_{on} the mass flowrate exiting the narrow control volume. The substance concentration is therefore $C_n = m_s/(m_{in} + m_s)$. Performing the same analysis to the wider section

$$m_{iw} + m_s = m_{ow}$$

where m_{iw} is the mass flowrate at the input of the wider section, m_s the mass flowrate of the substance entering the control volume and m_{ow} the mass flowrate exiting the wide control volume. The substance concentration is therefore $C_w = m_s/(m_{iw} + m_s)$. Since $m_{in} = m_{iw}$ we should get that $C_n = C_w$

In general, although I consider that the topic is of scientific importance, the mechanisms leading to the blood flow should be discussed in more detail, providing quantitative information, before the manuscript can be accepted.
