# Supplementary material for: On the driver of blood circulation beyond the heart
Source: PLoS One. 2023 Oct 19;18(10):e0289652. doi: 10.1371/journal.pone.0289652 (PMC10586597; doi:10.1371/journal.pone.0289652)
Supplement: Attachment — Submitted filename: Referee_Report.pdf [file pone.0289652.s009.pdf]

Report on manuscript # PONE-D-22-35747R1 "On the Driver of Blood Circulation Beyond the Heart" by Zheng Li and Gerald H. Pollack.

The manuscript has been revised and the authors have responded to my comments. I understand that "the scope of the present work is merely to demonstrate that blood vessels can use IR energy to transport blood, and to point to a plausible mechanism". However, I regret that the authors are reluctant to provide a more quantitative information of he mechanisms leading to the motion. A characteristic velocity induced by the gradients of concentration can be established based on the characteristic length scale and the diffusivity. Nevertheless, I understand the difficulties alleged by the authors and I would agree to accept the publication of the paper. I only have a minor comment that should not be difficult to clarify in the revised version.

Minor comments,

1. I am not sure to agree (or fully understand) the mechanisms discussed in the "Flow Direction: Model Considerations" section (Figure 4). I agree that the narrow section has a higher surface-to-volume ratio but I am not sure to understand that the flow should go from the narrow to the wide section. I would encourage the authors to include the analysis supported by the application of the mass conservation equation to control volumes in the narrow an the wide sections. In fact, if it is assumed that the surface activity results in substances entering the tube at the same rate per unit surface area throughout the length of the tube, a lower quantity of substance would enter the narrow section since the exchange area, πDL, is smaller than in the wide section. Please clarify the discussion in this section supporting the information with the governing equations.

In general, although I consider that the topic is of scientific importance, some of the mechanisms leading to the blood flow are not sufficiently clear and should be clarified before the accepting the manuscript.
