# Supplementary material for: On the driver of blood circulation beyond the heart
Source: PLoS One. 2023 Oct 19;18(10):e0289652. doi: 10.1371/journal.pone.0289652 (PMC10586597; doi:10.1371/journal.pone.0289652)
Supplement: Attachment — Submitted filename: Response to Reviewers, Round II.docx [file pone.0289652.s010.docx]

Response to Remaining Issues in Revision Round 2

“*On the Driver of Blood Circulation Beyond the Heart*”

Zheng Li, Ph.D. and Gerald Pollack, Ph.D.

Department of Bioengineering, University of Washington, Seattle, Washington, USA.

*Correspondence to: zhl@u.washington.edu, ghp@u.washington.edu

We thank the editor and referees for their careful review of our manuscript. We have now fully responded to the reviewers’ comments. Our responses are presented below.

**Response to Reviewer #1**

*The manuscript has been revised and the authors have responded to my comments. I understand that ”the scope of the present work is merely to demonstrate that blood vessels can use IR energy to transport blood, and to point to a plausible mechanism”. However, I regret that the authors are reluctant to provide a more quantitative information of he mechanisms leading to the motion. A characteristic velocity induced by the gradients of concentration can be established based on the characteristic length scale and the diffusivity. Nevertheless, I understand the difficulties alleged by the authors and I would agree to accept the publication of the paper. I only have a minor comment that should not be difficult to clarify in the revised version.*

*Minor comments,*

*1. I am not sure to agree (or fully understand) the mechanisms discussed in the ”Flow Direction: Model Considerations” section (Figure 4). I agree that the narrow section has a higher surfaceto-volume ratio but I am not sure to understand that the flow should go from the narrow to the wide section. I would encourage the authors to include the analysis supported by the application of the mass conservation equation to control volumes in the narrow an the wide sections. In fact, if it is assumed that the surface activity results in substances entering the tube at the same rate per unit surface area throughout the length of the tube, a lower quantity of substance would enter the narrow section since the exchange area, πDL, is smaller than in the wide section. Please clarify the discussion in this section supporting the information with the governing equations.*

*In general, although I consider that the topic is of scientific importance, some of the mechanisms leading to the blood flow are not sufficiently clear and should be clarified before the accepting the manuscript.*

We thank Reviewer #1 for reviewing our manuscript and for the valuable feedback. We also wish to express our gratitude for bringing up the question about flow direction, as it is likely to be a concern shared by other readers. Addressing this question will therefore enhance the clarity of our work.

To reiterate, the driving force behind surface-induced flow should be the substance-concentration gradient, rather than the absolute quantity of the substance.

Consider a cylindrical tube with radius $R$ and length $L$, where substance $X$ can enter or exit the tube through its surface at a rate of amount $A$ per unit surface area per unit time. In this piece of tube, we define the concentration of substance $X$, denoted as $c_{X}$, as the quantity of the substance, $n$*,* divided by the volume of the tube, $V$. Assuming the initial substance concentration is $c_{X(t=0)}$, then at a given time interval $\Delta t$, the concentration of substance $c_{X}$ can be expressed as the sum of the initial concentration and the change of concentration:

$$c_{X}{=c_{X(t=0)}+\Delta c}_{X}=c_{X(t=0)}+\frac{\Delta n}{V}=c_{X(t=0)}+\frac{A\cdot2\pi R\cdot L}{\pi R^{2}\cdot L}\Delta t=c_{X(t=0)}+2A\Delta t\frac{1}{R}$$

Considering the model depicted in Fig. 4, where a narrow tube is connected to a wide tube, let us assume that substance $X$ enters the tube (e.g., *A* is positive) and the initial substance concentration is the same in both tubes. Based on the equation above, at a given time interval $\Delta t$, the concentration difference between the narrow section and the wide section is given by:

$${c_{X}}_{narrow}-{c_{X}}_{wide}=2A\Delta t\left( {\frac{1}{R}}_{narrow}-{\frac{1}{R}}_{wide} \right)<0$$

Thus, the substance concentration in the narrower section will be greater than that in the wider section.

Assuming the flow of the substance in the longitudinal direction is caused by diffusion, the direction of the flow should follow the direction of the concentration gradient - from high concentration to low concentration. Since the substance concentration is higher in the narrow tube, the direction of the longitudinal flow should be from narrow to wide.

We hope the above explanation has cleared up the flow-driving mechanism. We have included the above explanation in the supplemental material.
